# Why friendship and loneliness affect our health

**DOI:** 10.1111/nyas.15309

**Published:** 2025-03-06

**Authors:** Robin I. M. Dunbar

**Affiliations:** ^1^ Department of Experimental Psychology University of Oxford Oxford UK

**Keywords:** default mode neural network, Dunbar's Number, endorphins, friendship, loneliness

## Abstract

Humans, like all monkeys and apes, have an intense desire to be social. The human social world, however, is extraordinarily complex, depends on sophisticated cognitive and neural processing, and is easily destabilized, with dramatic consequences for our mental and physical health. To show why, I first summarize descriptive aspects of human friendships and what they do for us, then discuss the cognitive and neurobiological processes that underpin them. I then summarize the growing body of evidence suggesting that our mental as well as our physical health and wellbeing are best predicted by the number and quality of close friend/family relationships we have, with five being the optimal number. Finally, I review neurobiological evidence that both number of friends and loneliness itself are correlated with the volume of certain key brain regions associated with the default mode neural network and its associated gray‐matter processing units.

## Graphical abstract

Friendships play an especially important role in our lives, providing emotional and other sources of support as well as creating the communities on which our survival has depended. Friendship is underpinned both by core areas within the brain and by β‐endorphins. Because β‐endorphins have a number of direct beneficial effects on mental and physical health, loss of friendship and loneliness have significant adverse consequences.

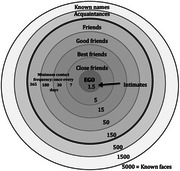

## INTRODUCTION

Like all monkeys and apes, humans are intensely social. Our collective success as a zoological family has depended on that sociality. However, sociality comes at a price. For mammals in general, group living is costly for two reasons. Living in close proximity to others creates stressors that quickly become overwhelming, triggering illness as well as psychological crises. More importantly, for mammals in general, these stressors have a dramatic impact on female menstrual endocrinology, leading very quickly to infertility.^1^ This decline is so steep that, all else being equal, mammals are unable to live in groups with more than about five reproductive females (the *infertility trap* ^1^).

A small number of mammalian species (notably anthropoid primates) have evolved the capacity to live in groups that are larger than five females in order to gain the benefits that large groups have to offer. In order to do so, they have had to find ways to mitigate the stresses of group living. In anthropoid primates, females do this by forming friendship‐based coalitions.^2^, ^3^, ^4^, ^5^, ^6^ Although alliances can be (and sometimes are) actively defensive, their main function is passive deterrence: simply persuading others to steer clear rather than risk a concerted counterattack by an alliance.^1^, ^7^ So long as others maintain a reasonable distance, stress is minimized and infertility held at bay. This is not a perfect solution, in that in all these species the infertility trap reappears eventually; nonetheless, coalitions defer the point at which the infertility trap reasserts itself sufficiently to allow females to live together in larger groups than they would otherwise have been able to do.^1^ The downside is that friendships are expensive to create and, especially, maintain, both in terms of the time that has to be invested in them and in terms of the cognitive abilities required to successfully navigate one's way through the social environment.

Most research on friendships has focused on a very small number of limited relationships, principally best friends (as in platonic friends, or “best friend forever”), romantic partners, or relationships with very close kin (a parent or sibling—what we might think of as friendships‐with‐kinship‐obligations). Very few have considered the wider picture of friendship circles (egocentric social networks), or asked how and why these might be limited in number. Even fewer have asked what functions friends perform for us (why we have friends). Among the few that have addressed the latter issue are Tooby and Cosmides^8^ and DeScioli and Kurzban,^9^, ^10^ while Fiske^11^ offered a promising early attempt to view friendships in a wider context. More recently, others have begun to address the nature of friendship in greater depth, providing an explicitly evolutionary framework for understanding some of the features of friendship and the way these differ between the sexes.^12^, ^13^, ^14^, ^15^, ^16^ In this contribution, I build on these studies and extend them by examining both the benefits that friends offer us and the neurobiology that underlies friendship formation as a way into explaining why the absence of friends leads to loneliness and adverse health consequences.

Forming and maintaining friendships involves the investment of a great deal of time as well as the ability to exercise the skills of diplomacy. The latter includes both mentalizing (understanding others’ intentions) and self‐control (the ability to inhibit prepotent actions).^17^, ^18^, ^19^ Inhibition allows individuals to coordinate their activities, as well as to avoid destabilizing relationships unnecessarily by pursuing their own short‐term selfish interests.^17^ Mentalizing is central to this: to avoid responding inappropriately to others’ actions, we need to understand their motives and intentions. Both mentalizing and inhibition are much more cognitively demanding than is often supposed, as well as involving dedicated neural systems in the brain (inhibition^7^, ^18^, ^19^; mentalizing^20^, ^21^, ^22^, ^23^, ^24^, ^25^). In the case of mentalizing, this involves one of the largest neural networks in the brain (the default mode network).^26^, ^27^, ^28^ Notwithstanding, many valiant attempts to claim that other mammals and birds have a primate‐like capacity for self‐control, the reality is that only anthropoid primates have the brain region that allows them to do this (the frontal pole, Brodman area BA10) and only they can pass genuine self‐control tasks.^18^ Without these two capacities, there is no incentive to hold back from pursuing one's own selfish interests, and group life would become effectively impossible.

The core to primate and human social life, then, is friendships—personalized relationships between two individuals based on a deep emotional bond.^17^ In anthropoid primates, the bonding process is created by social grooming. Social grooming, as I shall show below, triggers β‐endorphin release, and this in turn creates a deep sense of belonging. β‐Endorphins play a particularly important role in some of the ramifications of friendship and its antithesis (loneliness) that concern us here. Note that, in this respect, my use of the term “bond” is very different from that originally conceptualized by Bowlby^29^ in his concept of attachment. For Bowlby, attachment was specific to infant development and was an innate process (his *internal working model* concept) akin to the kind of instinctive imprinting that had been discovered by classical ethologists. My usage derives from social psychology^30^, ^31^: it is more general in its targets, involves two key dimensions (“feeling close” and “wanting to be close”), and is underpinned by an explicit pharmacological mechanism in the form of the endorphin system (reflecting what philosophers of mind would refer to as a “raw feels” process).

Before considering these aspects in more detail, however, let me first briefly review the size and structure of human social networks and the impact these have on our health and wellbeing.

## THE HUMAN SOCIAL WORLD

In primates, the species’ mean social group size is correlated with the size of its neocortex,^7^, ^32^, ^33^, ^34^, ^35^, ^36^ a relationship known as the social brain hypothesis. This relationship also seems to hold true in a few other unusually social mammals (cetaceans^37^; carnivores^38^), but is more generally present in the form of a difference between pair‐bonded versus herd‐forming species in all mammals and birds.^39^, ^40^, ^41^, ^42^, ^43^ The relationship between social group size and brain size also holds at the within‐species level: individual differences in egocentric network size correlate significantly with the size of the core brain areas responsible for managing social relationships in both humans^22^, ^44^, ^45^, ^46^, ^47^ and monkeys.^48^, ^49^, ^50^

The regression equation for these data predicts a natural grouping size for modern humans of ∼150 (± some error variance).^7^, ^51^ A large sample of natural human groupings, including data from hunter−gatherer societies, historical village societies, the sizes of business organizations, and the size of personal social networks (the latter based variously on data from face‐to‐face contacts, telephone call logs, Facebook and other social media, and email networks), yield an average grouping size of 154 (with a variance similar to that predicted by the primate regression equation).^51^

**FIGURE 1:**
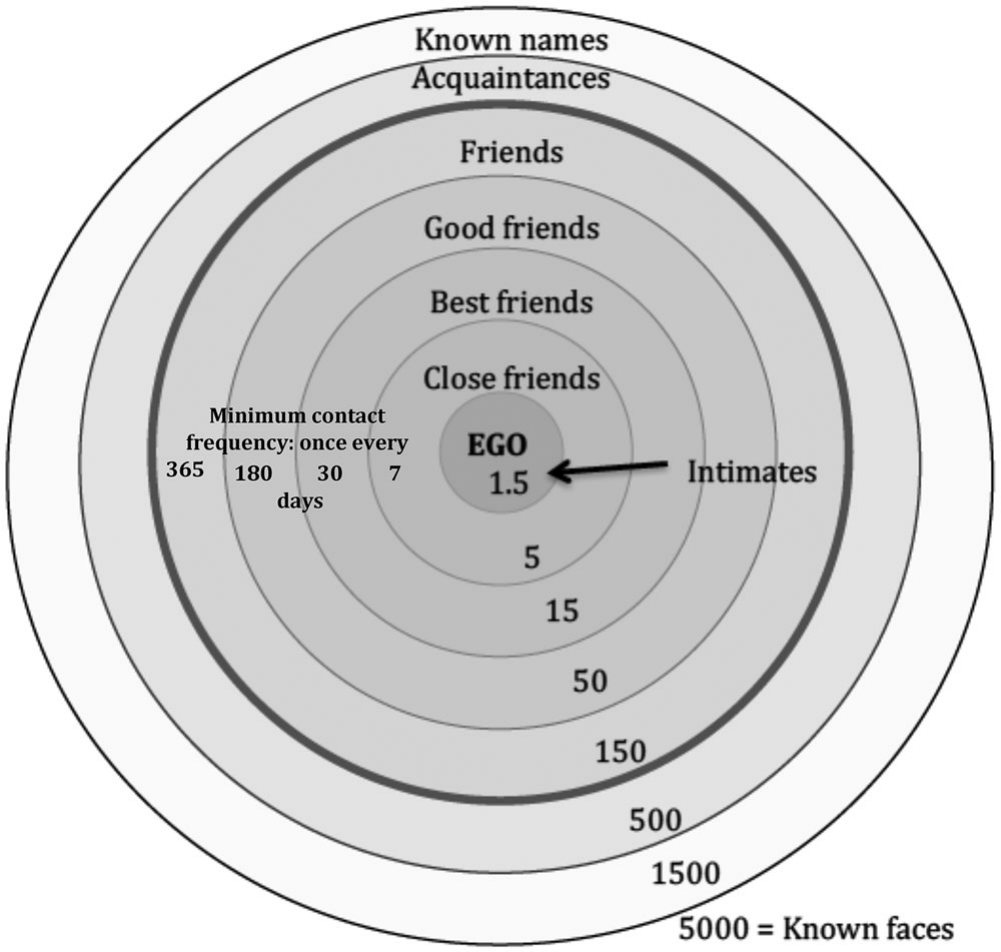
Circles of friendship, or Dunbar graph.^52^ Schematic diagram of the layering present in human ego‐centric social networks, illustrating the typical sizes (number of individuals) in each layer and the characteristic relationships involved. The “Friends” layer of ∼150 demarcates the limit on the number of meaningful relationships (those that are reciprocated, have a history, and involve relationships characterized by obligation, altruism, reciprocity, and trust). The layers within the 150 include both extended family and conventional friends. Time spent interacting with individual members, and willingness to act altruistically toward them, decrease across successive layers. Beyond the 150‐layer, relationships become more explicitly transactional, involve negligible social interaction, and are based on identity recognition rather than intimacy. Beyond the outermost layer at 5000, there are only strangers. *Sources*: Dunbar,^51^ Sutcliffe et al.^55^

This natural grouping size is, however, actually part of a series of numbers (a Dunbar graph^52^) with a very distinctive structure that has been identified both in the human data sets^40^ and in the group size distribution and internal structuring of primate social groups.^53^ These numbers form a series of layers at 1.5 (effectively, intimate friends), 5 (close friends), 15 (best friends), 50 (good friends), 150 (“just friends”), extending beyond the 150 layer in humans to include layers at 500 (acquaintances), 1500 (familiar faces), and 5000 (known faces) (Figure 1). Note that these layers count cumulatively: the 15‐layer includes the 5‐layer, and the 50‐layer includes the 15‐layer, and so on. This sequence has a natural fractal structure with a scaling ratio of ∼3, with each layer being three times the size of the layer immediately inside it. The layer sizes and their scaling are probably a consequence of the fact that these specific numbers (at least up to the 500‐layer) turn out to be optima that maximize information flow around social networks,^54^ such that they act as attractors around which group sizes converge.

The layers within the 150‐layer (the *natural* human grouping) are based on personalized relationships and deep historical knowledge,^55^, ^56^ with each layer corresponding to a particular level of familiarity, frequency of contact, emotional closeness, trust, reciprocity, and willingness to behave altruistically.^57^ Beyond the 150‐layer, relationships become increasingly transactional and anonymous. Beyond the outermost layer at 5000, everyone is a stranger: we do not even recognize their faces. An additional important consideration is that the personal network of 150 is broadly divided into extended family and true friends. In the modern, post‐demographic‐transition world, friends and family each make up about half of the network in each layer.^58^, ^59^ We give priority to family (the *kinship premium* ^57^), such that there is a negative relationship between the numbers of family versus friends in a network: people who come from large extended families have fewer friends.^58^, ^59^ In human social terms, this size of community would encompass an extended family out as far as second cousins and their offspring.^60^ Indeed, no language has kinship terms for individuals who are less closely related. The distinction between family and friends is important in that family relationships are relatively robust, but friendships decay very quickly if they are not invested in sufficiently.^61^, ^62^, ^63^ Rates of turnover tend to be low in the innermost 5‐layer (around one person being replaced every 10 years^64^), but are much higher in the other layers (where up to 30% of network members can change layer, or drop out altogether, each year^65^, ^66^).

What ultimately sets the limit on the size of the 150‐layer circle is that friendships are costly to maintain. Each layer identifies with a very specific frequency of contact if the relationship is to remain stable—once a week for the 5‐layer, once a month for the 15‐layer, and once a year for the 150‐layer^55^ (Figure 1), with these values being minima, not averages. Mode of contact seems to be unimportant, since we find the same clustering patterns with the same frequencies of contact from face‐to‐face interactions, phone calling, texting, and postings on social media.^67^ Failure to meet up sufficiently frequently causes the emotional closeness of a friendship to decline measurably within just a few months,^60^, ^62^, ^63^ although it probably takes about 3 years of no contact for someone from the inner circles to slip out of the 150‐network altogether into the layer of acquaintances beyond. There seems to be an intuitive appreciation of this since, at least in respect of phone calls, failure to call at the expected rate results in a significant increase (by about 50%) in the length of the next call to that individual as though endeavoring to repair a weakened relationship.^68^

One important aspect of friendships is the stark difference in the social styles of the two sexes. In simple terms, men's friendships are rather anonymous and clublike, whereas women's are more dyadic and personalized.^69^, ^70^ Men's friendships tend to be friendships of convenience; they are more substitutable and easily replaced, forming casually and decaying easily when individuals become geographically separated or simply drift apart through want of contact. Women's friendships, in contrast, tend to be more dyadic and focused, with who you are being more important than what you are. Like women's romantic relationships,^71^ they are typically more carefully selected and emotionally deeper; elaborate efforts are often made to maintain the relationship if the pair becomes separated. Women's friendships involve more complex cognitive processing,^72^ depend on different brain areas^73^ and are described as being more fragile (i.e., liable to catastrophic break‐up) because they are more demanding.^74^, ^75^ This difference in social style may explain the striking sex difference in the adverse consequences of loneliness (see below).

## FRIENDSHIPS WITH BENEFITS

**FIGURE 2:**
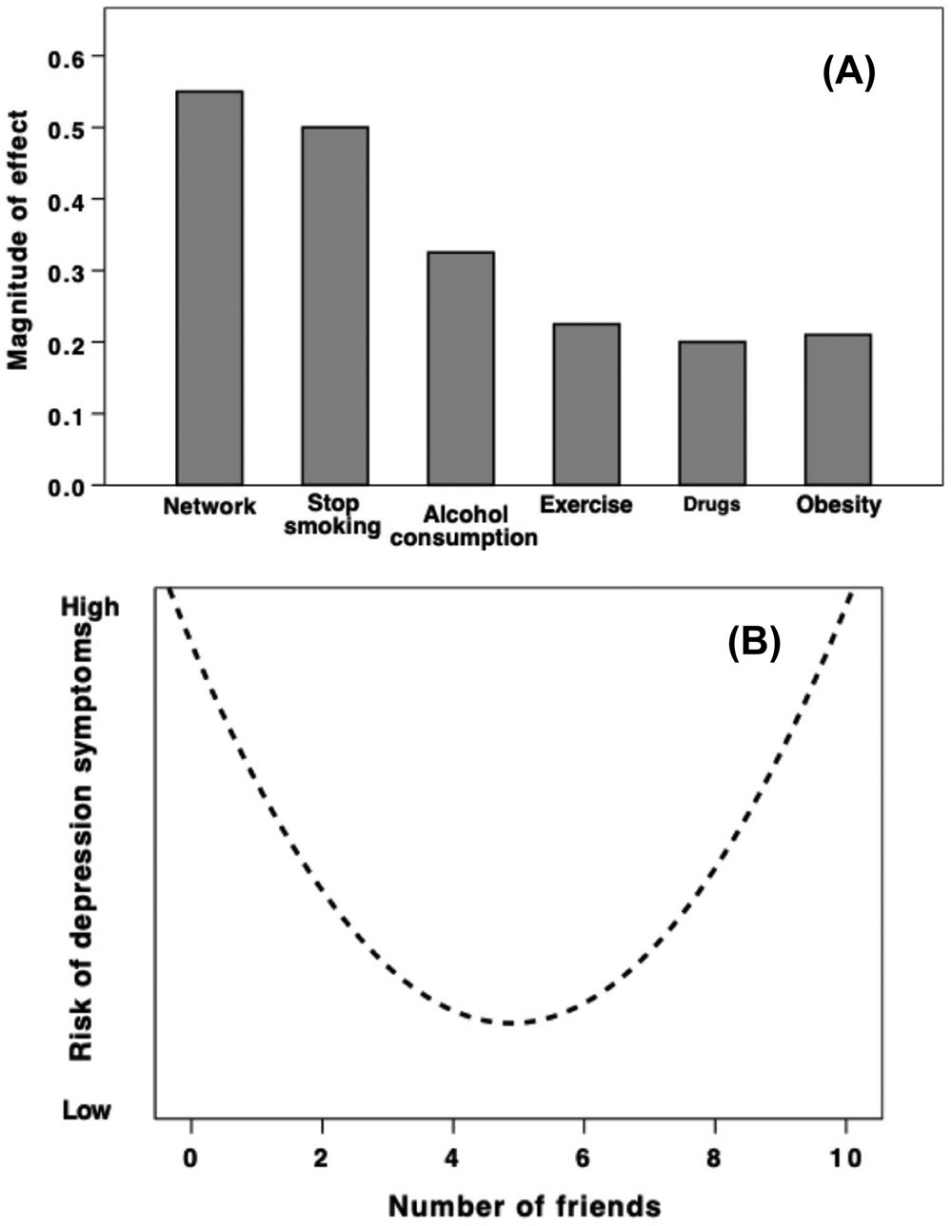
(A) Effect sizes for the influence of different factors on the likelihood of surviving for 12 months after a heart attack, based on a meta‐analysis of 148 epidemiological studies (involving 310,000 patients). Redrawn from Holt‐Lunstad et al.^83^ (B) Likelihood of developing symptoms of depression within 2 years as a function of the number of friends at the start, based on a prospective sample of 38,300 individuals (aged >50 years) from 13 European countries. Redrawn from Santini et al.^92^

The last two decades, in particular, have witnessed a veritable deluge of largescale surveys and prospective longitudinal studies showing that the single best predictor of mental health and wellbeing, physical health and wellbeing, and even longevity (survival into the future from now),^76^, ^77^, ^78^, ^79^, ^80^, ^81^, ^82^, ^83^, ^84^, ^85^, ^86^, ^87^, ^88^, ^89^, ^90^, ^91^, ^92^ as well as economic prospects,^93^ is simply the number and quality of close friends and family. Many of these studies involve very large samples (*N*>5000 individuals). Consistently, the optimal number of friends to maximize health and wellbeing is five. Figure 2 illustrates this with data from two such studies. One is a meta‐analysis of 148 epidemiological studies of heart attack patients (*N* = 310,000 individuals)^83^; the other is a prospective study of symptoms of depression and life satisfaction (*N* = 38,000 individuals).^92^

The meta‐analysis by Holt‐Lunstad et al.^83^ is important for two reasons aside from the size of its sample. One is that its outcome variable (whether or not a patient survived for 12 months after their first heart attack) is especially hardnosed compared to the usually vague self‐rated happiness scores of many studies. The second is that it included a wide range of other variables as potential predictors: in addition to the number and quality of close friends, these included tobacco and alcohol consumption, diet, exercise regime, body mass index, local air quality, and prescribed medicines. Although most of these variables predicted survival to some degree, two variables, in particular, stood out as having much stronger effects than any of the others: the number and quality of close friendships and tobacco consumption (Figure 2A).

The prospective study by Santini et al.^92^ is of interest not just because it related the change in symptom state at the end of the study to the individuals’ social circumstances 2 years earlier, but also because it identified both the number of friends an individual had and whether or not they were involved in volunteer activities (e.g., helping with the scouts or a political party, running a club, or looking after the flower arrangements at church). As Figure 2B indicates, the likelihood of displaying more symptoms of depression was a ∪‐shaped function of the number of friends. It was also a ∪‐shaped function of the number of volunteer activities. Conversely, the likelihood of being more satisfied with life was ∩‐shaped function of the number of friends and number of volunteer activities. The significance of volunteer activities is, of course, that they offer opportunities to interact with other volunteers, perhaps even to socialize with them outside of “work”. While the optimal number of friends was five and the optimal number of volunteer activities was three, these could be mixed‐and‐matched but not added together. In other words, having, say, three friends and a volunteer activity was as good as having five friends and no volunteer activities, or three activities and no friends, but having five friends and three activities left one in as bad a state as having seven friends or five volunteer activities. The reason seems obvious: volunteer activities are social, and both these and friendships involve considerable time investment in socializing. If you do too many activities, or have too many close friends, the time you can devote to each person involved is proportionately reduced, given that the time available for socializing is limited.^94^ As a result, the *quality* of the individual friendships is poorer than if one has fewer friends or activities. In other words, having too few friends is as bad as having many weak friendships because both mean not having sufficient social back‐up (not least because there are too few people to share the load^55^).

In an exemplary series of studies, Christakis and collaborators analyzed data from a multi‐wave longitudinal study of a single community over several decades (the Framingham Heart Study) to determine whether friends influenced each other's behavioral states.^81^ In a series of publications, they showed that people's future risk of giving up smoking, developing obesity, becoming happy or depressed, falling prey to various diseases, and even the likelihood of dying were all strongly influenced by the state of their best friend in the previous wave. If your best friend developed obesity or lost weight, you were more likely to do the same by the next wave. They were able to detect a signal for this effect as far out as the third‐step friend (friend of a friend of a friend), but beyond that the risk was essentially uncorrelated. These data do not tell us about the size of the close friendship group (e.g., the 5‐layer), but they do suggest that the effect is limited to quite a small circle (certainly not beyond the 15‐layer in Figure 1).

A recent study of a national UK sample of adolescents found that the optimal number of friends that maximized both mental health and educational performance was five.^95^ Having too few or too many friends correlated with poorer mental health and cognitive performance, as well as smaller core brain regions associated with the social brain (see below). In a smaller‐scale experimental study, Gigli et al.^96^ found that enforced social isolation during the COVID‐19 lockdowns resulted in a significant increase in anhedonia (reduced experience of pleasure and/or motivation to engage in pleasurable activities).

Luo et al.^86^ used data from a 6‐year prospective study of individuals over 50 years of age to assess the impact of loneliness as measured by the Revised UCLA Loneliness scale and a range of biological and contextual variables on mortality. They found that feeling lonely was a significant predictor of future symptoms of depression, functional limitations (e.g., difficulties in walking or picking up a coin), and to a lesser extent self‐rated health, leading to an average 14% increase in actual mortality over a 6‐year period—even after controlling for existing social relationships, age, sex, existing health conditions, income, education, marital status, and geographical proximity to family or friends.

In sum, close friendships are not simply pleasurable and a willing source of volunteers with whom to engage in socially pleasurable activities, but a crucial part of our armory against both mental and physical ill‐health. More importantly, our wellbeing and health are maximized by having a very specific number of friends (five)—although it should be noted that this optimum number does vary as a function of an individual's gender and personality (slightly more than five for women and extraverts, slightly less than five for men and introverts, with the differences being consistently statistically significant).^59^, ^63^, ^97^, ^98^

It should be noted that we are not alone in benefitting from friends in this way. Evidence from long‐term field studies of a number of intensely social mammals (chimpanzees, baboons, feral horses, zebra, dolphins) demonstrates that females with more social partners experience less harassment, have reduced stress levels in response to social upheavals, recover faster from injury, have higher fertility and more offspring that survive to adulthood, and more grandchildren.^99^, ^100^, ^101^, ^102^, ^103^, ^104^, ^105^, ^106^, ^107^, ^108^, ^109^

## THE PSYCHOPHARMACOLOGY OF FRIENDSHIP

Given the importance of friendship for health, the obvious question is what mediates this? Why do friendship and loneliness have these effects on our psychological and physical health and wellbeing? The answer seems to lie mainly with β‐endorphins.^110^, ^111^, ^112^ β‐Endorphins are neurotransmitters that are part of the opioid system and are widely active in both the central and peripheral nervous systems. Of all of the opioid peptides, β‐endorphins are the most important for the present purpose: they interact with, and reduce the activity of, the hypothalamic‐pituitary‐adrenal axis that regulates the body's stress and homeostatic responses,^113^ and are involved in regulating consummatory reward (including the reward of social interaction), parturition, hypothermia, respiratory depression, digestion, and the psychological experience of affect.^113^ In addition, they turn out to have impressive analgesic properties: weight‐for‐weight, β‐endorphins are 20–30 times^114^, ^115^ (or, by some estimates, even 100 times^116^) more powerful as analgesics than morphine.

β‐Endorphins do not cross the blood‐brain barrier, and peripheral and central (brain) β‐endorphin release are, therefore, largely (though not entirely) separate.^117^, ^118^ Perhaps because of this, they seem to behave rather differently in the two subsystems: in the brain, they behave like neurotransmitters, but in the peripheral system, they behave more like endocrine hormones. In the brain, β‐endorphins have a particular affinity for μ‐opioid receptors (MORs) just as morphine does, producing very similar analgesic effects. MORs are widely distributed throughout most of the brain, where they are involved in the management of physical and emotional stress and pain. Activation of MORs by β‐endorphins in the brain lowers feelings of stress and creates a sense of physical warmth,^119^ calmness and relaxation, of being at peace, of self‐other immersion, that all is well with the world.^111^, ^112^ Through this, they seem to be involved in creating a sense of trust and bondedness (though we do not know exactly how this is achieved). Abnormalities in endorphin function have been associated with a wide range of psychological disorders ranging from autism to personality disorders.^111^, ^120^

**FIGURE 3:**
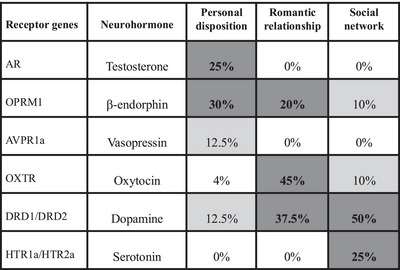
Heat map for the percentage of alleles for the receptors of each of the main social neurochemicals that correlated significantly with ratings on a variety of indices of social engagement at three different levels (social predisposition, dyadic romantic relationships, and total social network). The cells are color‐coded for magnitude (i.e., darker colors equate to higher magnitudes). Redrawn from Pearce et al.,^124^ corrected as noted by Pearce et al.^125^

The release of β‐endorphins in these contexts is also associated with the upregulation of dopamine neurotransmission,^121^, ^122^, ^123^ and the two neurohormones seem to work together in tandem in a social context. A large‐scale study of 33 single nucleotide polymorphisms (SNPs) for the receptors of the six neurohormones most commonly associated with social behavior (testosterone, β‐endorphins, oxytocin, vasopressin, serotonin, and dopamine) revealed that it was SNPs in *OPRM1* (the gene that codes for MOR) and *DRD2* (the gene that codes for the dopamine D2 receptor) that were principally associated with both social predisposition and social engagement (social network size and bonding), with the role of oxytocin receptors largely confined to romantic relationships (in addition to their principal roles of facilitating parturition and regulating milk let down during lactation) (Figure 3).^124^, ^125^

**FIGURE 4:**
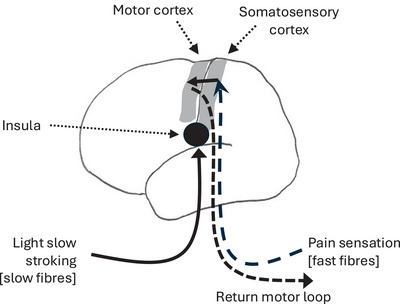
The afferent C‐tactile pathway (left side) contrasted with the more conventional peripheral pain/motor system (right side).

The principal activity that stimulates the release of central β‐endorphin in primates is social grooming. In most social monkeys and apes, social grooming can occupy as much as 20% of the day.^126^, ^127^, ^128^ Grooming acts on the brain's endorphin system via highly a specialized component of the peripheral nervous system, the afferent C‐tactile (CT) nerves. These C‐class neurons are activated by low threshold mechanoreceptors distributed throughout the hairy skin (i.e., everywhere except the soles of the feet and the palms of the hands).^129^, ^130^, ^131^, ^132^, ^133^ They respond only to skin deformation when the hand moves across the fur. These CT neurons are very unusual: in contrast to all other peripheral nerves, they are unmyelinated (and hence have very slow transmission rates), have no return motor loop, target the insula instead of the somatosensory areas in the brain (as most other peripheral nerves do), and respond to one stimulus and one stimulus only (light, slow stroking at approximately 3 cm per second [cps]—about the speed of hand movements across the fur during grooming)^134^, ^135^, ^136^ (Figure 4). This last characteristic is extremely restricted: stroke someone at ∼3 cps and their CT neurons will be activated; stroke them at 1 or 30 cps and nothing will happen.^137^

**FIGURE 5:**
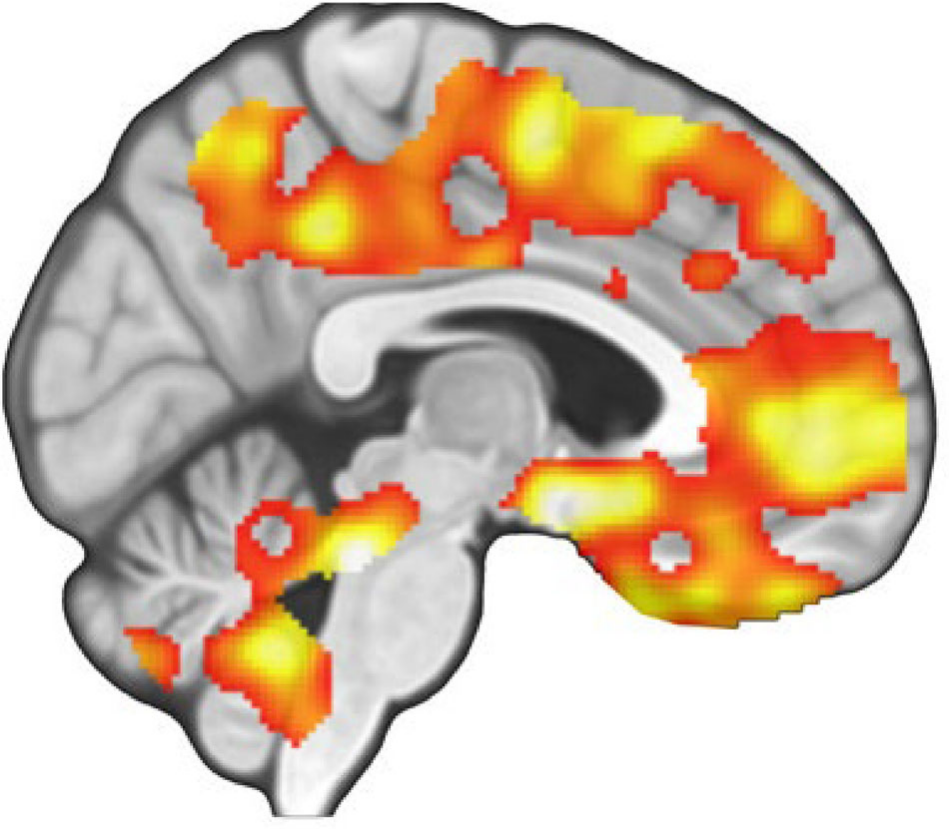
Activation of μ‐opioid receptors in the human brain while being stroked on the torso during a PET imaging experiment. Red and yellow sectors indicate where μ‐opioid receptors for β‐endorphins were differentially activated by the social touch. Reproduced from Nummenmaa et al.^139^

That it is specifically β‐endorphins that are released during grooming was originally demonstrated by Keverne and collaborators.^138^ They showed that when monkeys were given morphine (a MOR agonist), they lost interest in grooming, but when given naloxone (an opiate receptor antagonist that blocks MOR and prevents β‐endorphins binding), the monkeys constantly requested grooming. Later, Nummenmaa et al.^139^ used positron emission tomography (PET) imaging in humans to show that being stroked on the torso by a romantic partner results in a modulated MOR response over much the brain (Figure 5).

β‐Endorphins generate many of the health benefits we noted in the previous section, both directly and indirectly. The indirect effects come about through the way β‐endorphins help form bonded friendships: friends are then willing to support us when we are down and/or offer help when we need it out of a sense of obligation. The direct effects come via two distinct routes. Opioids naturally lift the mood and reduce psychological stress (the opioid high). Since, unlike more conventional opiates, β‐endorphins are not physiologically addictive and we do not habituate to them in the way we do to morphine and other opiates,^140^ the uplift they give when activated organically by social interaction may be the best antidote available for depression.^120^, ^141^, ^142^, ^143^, ^144^ The second is that a byproduct of β‐endorphins is that they activate the immune system.^145^, ^146^, ^147^, ^148^, ^149^ Of particular relevance is the fact that they upregulate the natural killer cells that target viruses and some cancers, as well as activating other cytotoxic lymphocytes.^150^, ^151^, ^152^, ^153^ This would seem to provide a direct mechanistic explanation for the fact that having friends—or, at least, doing β‐endorphin‐stimulating activities with them—can have such a dramatic effect on physical health.

**FIGURE 6:**
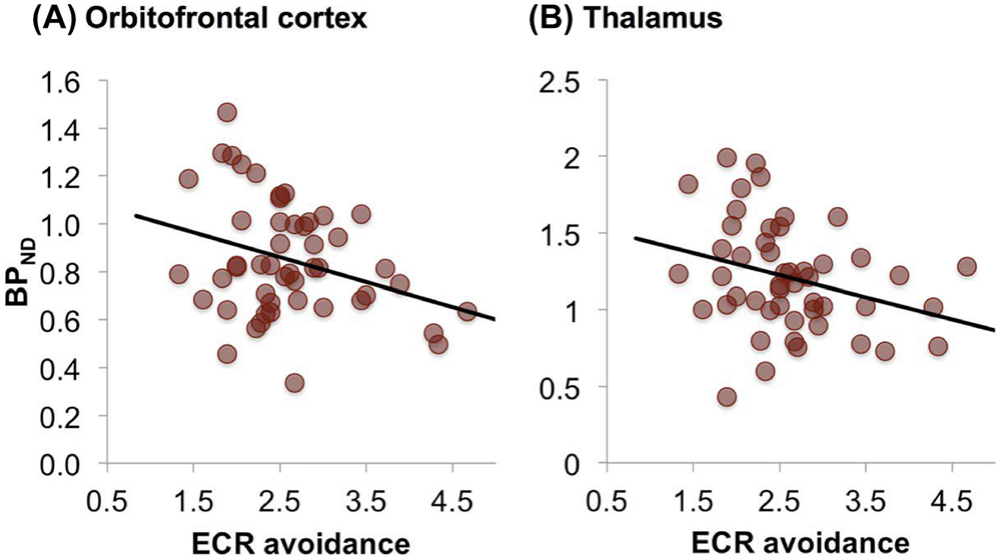
Relationship between avoidant attachment style and μ‐opioid receptor density in (A) orbitofrontal cortex and (B) thalamus, representing the reward and pain circuits, respectively (*p*<0.05 in both cases, false discovery rate corrected) in a human PET imaging experiment. Abbreviations: BP, binding potential; ECR, Experiences in Close Relationships scale. Reproduced from Nummenmaa et al.^155^

**FIGURE 7:**
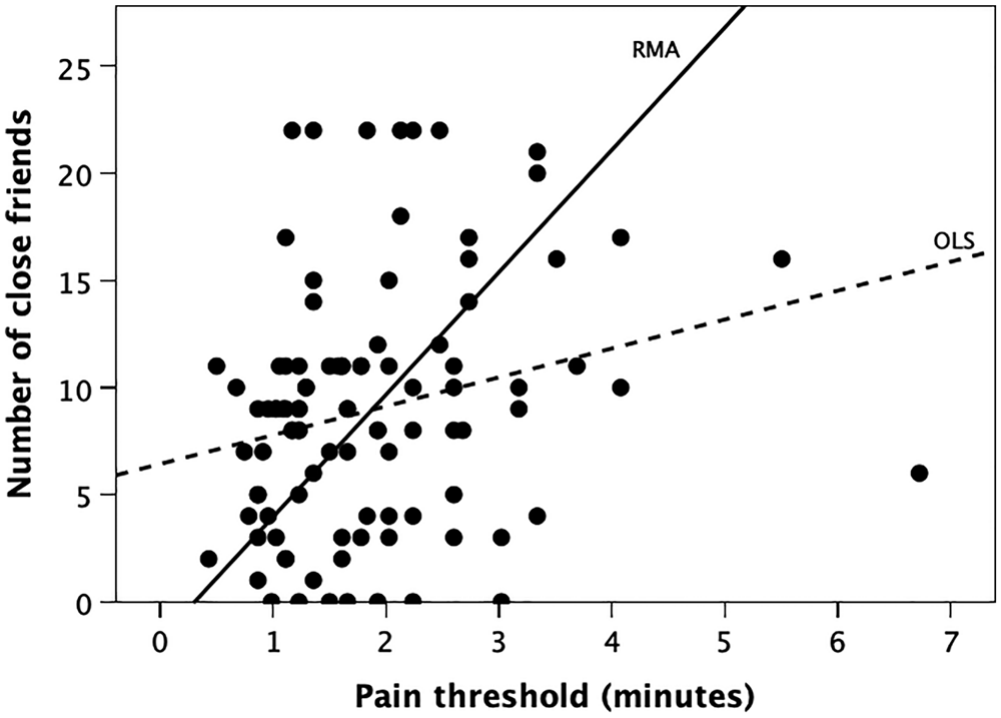
Number of close friends plotted against basal pain threshold (indexed as length of time, in seconds, for which a stressful skiing exercise known as the Roman chair could be held). The OLS regression (dashed line) is significant (*r* ^2^ = 0.06, *F*_1,96_ = 5.7, *p* = 0.019; with log_e_‐transformed pain threshold: *F*_1,96_ = 7.3, *p* = 0.008). Since there is considerable error variance on the X‐axis, OLS regression will radically underestimate the true slope unless *r* ^2^>0.95; in these circumstances, an RMA regression (solid line) gives a better estimate of the true slope.^35^ Abbreviations: OLS, ordinary least squares regression; RMA, reduced major axis regression. Redrawn from Johnson and Dunbar.^156^

One final observation on the importance of endorphins in regulating our social world is provided by a study that showed the density of the avoidance dimension of the Experiences in Close Relationships^154^ attachment scale was negatively correlated with MOR density in the thalamus, the cingulate cortices, the medial and lateral prefrontal cortex, the ventral striatum, insula, and amygdala (Figure 6).^155^ In contrast, the anxiety dimension of the attachment scale exhibited no significant correlations with any brain regions of interest. In effect, people who score high on the avoidance scale (i.e., have a rather cold, withdrawing attachment style) have low MOR density in key areas associated with the management of relationships (medial and orbitofrontal cortex). These results mesh with those from a study by Johnson and Dunbar^156^ that found that people's inner core social network size (the 5‐layer) correlated with their basal pain tolerance (Figure 7). The more pain an individual was able to tolerate, the more close friends they had.

One interpretation of these results is that individuals with low receptor density find their available receptors satiated after just a few social interactions, and so withdraw from further interaction, thus limiting the size of their social network. This may explain why introverts typically have smaller friendship circles than extraverts.^97^ Taken together, these results remind us that individuals can vary considerably in these traits (albeit within well‐defined limits), and these differences are likely to have significant effects on the propensity to feel lonely and the health and wellbeing consequences that follow.

## THE HUMAN SOCIAL TOOLKIT

**FIGURE 8:**
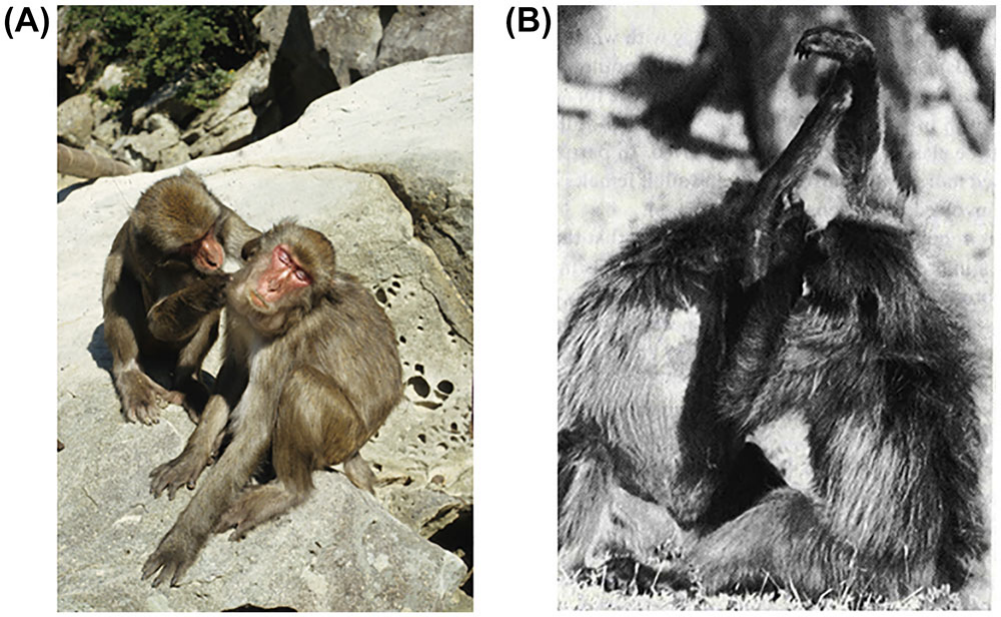
(A) Japanese macaques grooming. As is often the case when monkeys are being groomed, the recipient has dropped off to sleep. (Photo R. I. M. Dunbar) (B) Crossed‐arms grooming in gelada baboons, in which each individual simultaneously grooms the partner's forearm. (Photo R. I. M. Dunbar).

As a mechanism for bonding relationships, social grooming suffers from two practical disadvantages. First, it is normally unidirectional (i.e., only one animal can groom at a time, while the recipient often drifts off to sleep) (Figure 8A). As a result, monkeys and apes take it in turns to groom and be groomed—which they typically do alternately at roughly 5‐min intervals. Thus, 10 min engaged in grooming actually represents just 5 min being groomed (i.e., having one's β‐endorphin system up‐regulated) since the groomer itself does not gain any β‐endorphin hit. By adapting the hand movements of grooming in the way humans have done during caressing, we are able to “groom” each other simultaneously, effectively doubling the amount of grooming time. Some monkeys and apes occasionally groom each other simultaneously, but compared to humans, this is rare (never more than 10–15% of time spent grooming^129^) and has only been observed in a few populations of chimpanzees and baboons, where it is largely confined to the forearms (Figure 8B). Second, grooming is strictly dyadic (i.e., normally involves only two individuals grooming each other). Again, the more social monkeys and apes do occasionally engage in forms of group grooming,^129^ but this is very much the exception rather than the rule, even in humans.

**FIGURE 9:**
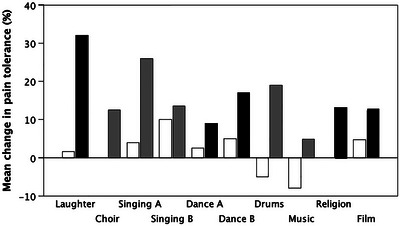
Mean change in β‐endorphin release (indexed as change in pain tolerance from before to after activity) as a consequence of engaging in different activities. Black and gray bars: experimental activity; white bars: control condition. Black bars indicate studies where β‐endorphin release was confirmed, either by PET‐imaging or by naltrexone administration. Control conditions varied with activity (e.g., neutral video, nonsinging hobby class, gentle arm‐movements while seated, listening to music rather than playing). Laughter (total of seven experiments: six in UK, one in Netherlands); Singing A and B (two separate experiments, one in UK and one in Netherlands); Dance A and B (two separate experiments, one in UK and one in Brazil); Music (listening to music with or without active head‐nodding); and Film (tragedy video vs. factual documentary) were carried out in the lab; one of the laughter experiments and the Choir and Drums (drumming circle vs. passive listening) studies were carried out in natural settings (all in the UK). Pain tolerances were determined using the cold pressor task (frozen sleeve or ice bucket), cuff sphygmomanometer, or wall‐sit task (Roman chair), all indexed by duration.^162^, ^164^, ^165^, ^166^, ^167^, ^168^, ^169^, ^170^, ^173^, ^174^, ^175^, ^176^, ^179^, ^180^, ^181^, ^182^ Redrawn from Dunbar.^199^

These constraints, combined with inevitable limits on the time that can be devoted to grooming each day,^157^ set an upper limit on the size of group that can be bonded by this mechanism.^129^ This limit seems to be set at about 50 individuals,^129^ even in humans.^158^, ^159^ In order to break through this threshold so as to increase community size, we humans have exploited a suite of behaviors that trigger the opioid system without requiring physical contact. These include laughter (as a form of chorusing), singing (with or without words), dancing, feasting (eating communally), and, once fully modern language had evolved, emotional storytelling, jokes, and the rituals of religion. We have shown, in an extended series of experimental and neuroimaging studies, that all these activities trigger MORs (Figure 9) and generate an enhanced sense of bonding (including synchronized activities like team rowing^160^ or social jogging; laughter^161^, ^162^, ^163^; singing^164^, ^165^, ^166^, ^167^, ^168^; music^169^, ^170^, ^171^, ^172^; dancing^173^, ^174^, ^175^, ^176^; feasting^177^, ^178^; storytelling^179^; and the rituals of religion^180^, ^181^, ^182^). The important feature of this is that the bonding process dramatically affects the individuals who do the activity together, but has no effect at all on the quality of existing relationships (even for best friendships) if these individuals are not present at the time.^174^

## LONELINESS AND THE BRAIN

With this background on the nature of human friendships and their function, we can now consider what happens when we are prevented from seeing our friends and relations. Fortuitously, the COVID‐19 pandemic and resulting lockdowns provided us with a large‐scale natural experiment in which people's everyday social lives were highly disrupted by enforced social isolation. The psychological consequences included elevated levels of anxiety and depression, a deterioration in mental health, changes in eating habits, increased suicidal ideation, and higher levels of experienced loneliness.^183^, ^184^, ^185^ These effects were by no means uniform, however. While some people experienced some or all of these symptoms, others experienced few adverse consequences. Not too surprisingly, perhaps, loneliness was highest among those who were forced to self‐isolate alone and who were unemployed, and was lowest among married or cohabiting couples (who obviously at least had one other person to talk to).^186^ An analysis of data from ∼10,000 adults in a nationally representative UK sample (the UK Household Longitudinal Study) found much greater increases in psychological distress among women and young adults, as well as in the Asian community and those who had had a university education, than in any other cohorts.^187^

In another UK study of >7000 cognitively healthy older adults, around 12% of individuals reported increased symptoms of anxiety and depression as a result of lockdown, often associated with sleep disturbances.^188^ Those who felt lonely prior to COVID had a 10–15 times higher odds of feeling more anxious or depressed during and after the lockdown compared to those who had not felt lonely. Being single, widowed, or divorced was a risk factor for poorer mental health during COVID in cohort studies from the UK, Spain, and China.^188^ A meta‐analysis of 65 longitudinal studies from North America, Europe, and elsewhere revealed an increase in depression, anxiety, and mood disorder symptoms.^189^ Several of these changes were especially pronounced in individuals with pre‐existing physical conditions (possibly the result of an elevated risk of infection and the negative effect this inevitably has on mood). An analysis of data for >5000 adults from the English Longitudinal Study of Aging found that depression, anxiety, loneliness, and a poorer quality of life all increased in the months after the pandemic, with these effects being stronger in women, older individuals, those living alone, and those less affluent.^190^

One consistent finding in all these studies is that women experienced worse effects than men, an observation replicated in different age groups and in countries ranging from the UK, Demark, Spain, and Italy to Turkey and Iran.^188^ Women who reported being lonely before the pandemic were twice as likely as lonely men to report an increase in symptoms of depression during the pandemic; in contrast, lonely men were only around 5% more likely to report increased levels of anxiety compared to women. Interestingly, online contact with friends or family did not significantly alter the risk of feeling depressed, and, indeed, in some studies made it worse,^184^ confirming earlier findings suggesting that online environments are less socially satisfying than face‐to‐face interaction.^191^ Best et al.^192^ also found that, compared to men, women experienced more overall distress and higher levels of panic, depression, emotional disturbance, and concerns about contracting COVID‐19. These sex/gender differences are in line with the fact that women's friendships are more emotionally close.^70^

One brain‐imaging study compared macaque monkeys housed in social isolation for 1.5 years with a subsequent period when they were housed socially with other monkeys.^193^ The repercussions of diminished frequency and intensity of social interaction were traced to the dopamine D2 receptors that are implicated in reward processing (although the study only considered receptors in the basal ganglia as they were mainly interested in the implications for motor coordination). Indeed, after social rehabilitation, less socially interactive males with fewer grooming opportunities displayed hyperactive dopamine responses not present before the isolation condition. The authors concluded that experimentally altering the social richness in the environment led to reward‐related neural plasticity. The medial‐temporal limbic system has also been implicated in plasticity changes in social network size in nonhuman primates.^48^

In humans, social isolation was negatively associated with the size of the prefrontal cortex, the association cortex, and the default mode neural network (DMN: the massive white matter connectome and the gray matter processing units it serves to connect).^28^, ^194^ The DMN is involved in processing information about oneself, about others’ thoughts and likely intentions, and the management of social relationships.^195^, ^196^ An analysis of data from the UK Biobank (*N*>40,000, aged 40–69 years) revealed stronger functional communication in the DMN among lonely individuals (particularly associated with the microstructure of the fornix pathway) and a decoupling of the DMN from the visual system. In addition, lonely individuals had significantly less gray matter volume in some frontal and parietal lobe regions as well as the visual system, and concomitantly larger gray matter volumes in some temporoparietal and temporal lobe regions. These observations would imply that those who are more socially active and/or have more developed sociocognitive skills may experience more loneliness when forced to isolate.

An analysis of 48 different white matter tracts revealed a particularly strong association between loneliness and the fornix, a core unidirectional neural tract responsible for transporting neural signals from the hippocampus to the medial DMN.^28^ The microstructure of this tract varies between individuals as a function of episodic memory, in particular the vividness of recall. The observed pattern suggests that loneliness may be associated with a shift of emphasis from integrative networks (e.g., connections within the DMN facilitating social information processing) to sensory networks (e.g., DMN connections to the visual system), with this shift being greater for men.

In the absence of further evidence, the causality in these findings remains uncertain. Do the observed anatomical patterns result from changes caused by isolation or is it that individuals who have these anatomical traits are predisposed to experience more adverse consequences from isolation? Since changes to brain region volume and connectivity take some time to take effect, and the pandemic lockdown periods were themselves quite short, the second causal sequence may be the more likely. These patterns might explain why lonely individuals often turn in on themselves psychologically, devoting more time to mentalizing, reminiscence, and imagination to fill a social void.^26^, ^28^ This can involve anthropomorphizing pets, building intense relationships with TV characters, and reminiscing about past social exchanges with people who had previously dropped out of one's social network. This is not always bad news, of course, since in some cases, these traits can be turned to creative advantage by those who have a flair for storytelling. Most people, however, are likely to experience severe distress.

The results summarized here make it clear that a functional social environment is important for humans, such that they are likely to experience adverse consequences at many different levels if prevented from socializing. Being denied the opportunity to meet and interact with friends even for relatively short periods of a few months will inexorably set in motion relationship decay,^61^, ^62^, ^63^, ^65^, ^66^ and this in turn is likely to have adverse consequences for both mental and physical health^190^ if friendships are lost and not replaced relatively quickly.

## CONCLUSION

Our extraordinarily intense form of sociality makes us particularly vulnerable to loneliness. Because of the role that β‐endorphins play in building and maintaining friendships and the direct and indirect effects these have on our health, social isolation exposes us to significant risks of mental and physical ill‐health. In effect, friends provide a natural remedy for depression and anhedonia. In this respect, as Cacioppo^197^ has argued, loneliness can best be understood as an evolutionary signal that all is not well with our social life and we urgently need to do something to remedy the situation. However, creating and maintaining friendships is time‐costly, and requires effort and persistence. If friendships are to alleviate depression and illness, they have to be established well ahead of need since it is the sense of trust and obligation established over many hours of dyadic interaction with a specific individual^198^ that impels us to respond positively to their requests for support. We help friends willingly out of a sense of personal obligation toward the individual concerned, but we help strangers only out of charity.^164^

## COMPETING INTERESTS

The author declares no competing interests.

### PEER REVIEW

The peer review history for this article is available at: https://publons.com/publon/10.1111/nyas.15309

## Data Availability

Data for individual graphs are available in the source publications.
